# Modulating the Structural, Thermal and Techno‐Functional Properties of Sesame Protein Isolate Using Nonthermal Techniques

**DOI:** 10.1002/fsn3.70144

**Published:** 2025-04-03

**Authors:** Osman Gul, Melike Seyda Sahin, Abdullah Akgun, Latife Betul Gul

**Affiliations:** ^1^ Department of Food Engineering Faculty of Engineering and Architecture, Kastamonu University Kastamonu Turkey; ^2^ Department of Food Engineering Faculty of Engineering, Trakya University Edirne Turkey; ^3^ Department of Food Engineering Faculty of Engineering, Ondokuz Mayıs University Samsun Turkey

**Keywords:** nonthermal techniques, physical modification, sesame protein isolate, techno‐functional properties

## Abstract

Sesame protein isolate is a promising sustainable plant‐based protein due to its high nutritional value and unique flavor. However, due to several challenges related to its functional characteristics, sesame protein can only be used sparingly in food applications. Therefore, the main objective of this study was to investigate the effects of nonthermal techniques including high‐pressure homogenization (HPH, 100 MPa), high‐intensity ultrasound (US, at a frequency of 20 kHz for 6 min), and high hydrostatic pressure (HHP, 400 MPa for 5 min) on the structure and functional properties of sesame protein isolate from sesame cake as a by‐product. The results indicated that all nonthermal treatments encouraged the sesame protein insoluble suspension to change into a consistent protein dispersion, increasing the stability of the protein while reducing particle size (from 65.73 to 1.48 μm), increasing zeta potential (from −24.57 to −42.8 mV), and unfolding the molecular structure. All treatments led to an increase in β‐sheets and reduced α‐helix, and the most remarkable change in secondary structure occurred in the HPH treated sample that exhibited the highest UV absorbance. Minimal impact on the protein's thermal properties was monitored. Compared with the untreated sample, the techno‐functional properties were significantly enhanced after modification, and the highest protein solubility (88.18%), EAI (62.09 m^2^/g), ESI (65.43 min), and OHC (1.89 g oil/g protein) obtained in the sample treated with HPH. Therefore, this work suggests that HPH could be a more promising technique than US and HHP to enhance the techno‐functional properties of sesame protein isolate.

## Graphical abstract

Sesame protein isolate from sesame cake was modified by HPH, US, and HHP. FTIR confirmed the reduction of α‐helix content and the increment of β‐sheet content in isolates. Thermal analysis indicated minimal impact on the protein's thermal properties. The techno‐functional properties of sesame protein isolate were greatly improved. HPH treatment gave optimal results for modifying isolates among all the treatments.

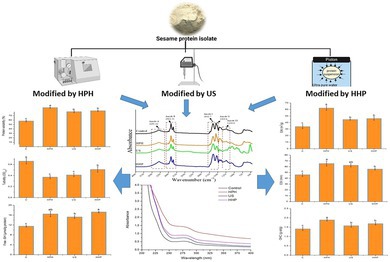

## 1 Introduction

Proteins, which are vital parts of human nutrition, are widely used macromolecules due to their functionality in food formulations and nutritional benefits. Because of their amphipathic structure, they are considered versatile structural components in food matrixes and serve as surface‐active agents due to protein‐water, protein‐air, protein‐carbohydrate, and protein‐lipid interfacial interactions (Akharume et al. 2021; Goyal et al. 2024). The presence of protein in food formulations mainly determines the overall quality by affecting the final product's food structure, texture, and sensory properties of the final product (Rathnakumar et al. 2023). The functionality of proteins attributes to their specific roles and properties in processes such as solubility, foaming, gelation, emulsification, water/oil‐holding capacity, viscosity, texture modification, etc., including interactions with other molecules (Karabulut et al. 2024). The primary typical source of protein in human nutrition is animal products. However, due to the high cost of animal protein, religious beliefs (halal food production), and the spread of vegan/vegetarian nutrition, plant‐based proteins have become more and more popular in recent years. Consumers' increased awareness and knowledge of food ingredients, their desire for clean and lean protein, and their interest in natural, eco‐friendly, and sustainable food sources have also contributed to this increasing trend. Another influential factor in increasing the demand for plant proteins is the recent interest in converting agricultural wastes containing valuable components like protein into high‐value‐added products. On this basis, the recovery of plant proteins from industrial food waste/by‐products has become an indispensable global necessity for the economy and society (Akharume et al. 2021; Kim and Shin 2022; Nikbakht Nasrabadi et al. 2021).

Literature has indicated that cereals (including wheat, corn, rice, maize, barley, and sorghum), legumes (including soybean, chickpea, mung bean, kidney bean, black bean, lupine, lentil, pigeon pea, peanut and cowpea), and oilseeds (including sesame, sunflower, canola, rapeseed, and cottonseed) are potential sources in the production of plant‐based protein. Additionally, many studies have demonstrated the potential of by‐products of cereals, legumes, and oilseeds (hazelnut, peanut, sunflower, sesame, etc.) as plant‐based protein sources for human nutrition and use as ingredients in food formulations (Kumar et al. 2022). Particularly in the oil industry, the remaining meals/cakes (also called oilseed press meals/cakes) after oil extraction have important potential as they have a protein content varying between 35% and 60% (Nevara et al. 2023). Sesame ( *Sesamum indicum* L.), which is one of the world's most crucial oilseed plants, is a potentially useful source of plant‐based protein. Organic solvents or the mechanical pressing process are used to extract sesame oil, and the extraction of sesame oil results in the production of sesame cake as a by‐product containing approximately 50% protein (Nevara et al. 2023). Sesame proteins have a very high potential for use as an ingredient in the food industry because they increase the nutritional value of foods (Yüzer and Gençcelep 2023). What distinguishes sesame protein from other oilseeds is its high methionine content (2.5%–4.0%), while sesame is insufficient in terms of lysine and isoleucine (Cano‐Medina et al. 2011). Sesame seeds are mainly composed of globulins (67.3%), albumins (8.6%), prolamins (1.4%), and glutelins (6.9%) (Achouri et al. 2012). In addition to its high nutritional value, it is also utilized as a food additive due to its techno‐functional properties such as emulsifying activity, foaming capacity, water/oil‐holding capacity, and whippability (Fathi et al. 2018). However, the use of sesame protein in food applications is limited due to difficulties associated with techno‐functional properties such as solubility, which directly affects other functional properties and digestibility (Di et al. 2022; Naeini et al. 2023). So, new approaches are needed to overcome this problem and expand the applications of sesame protein. In this context, recently, techno‐functionality and bioactivity have been improved by changing the molecular structure or several chemical groups of proteins with special methods, and accordingly, proteins have been turned into multifunctional components for food systems (Nikbakht Nasrabadi et al. 2021). Several innovative technologies, including high‐pressure homogenization (HPH), high‐intensity ultrasound (US), and high hydrostatic pressure (HHP), which are known as green food processing technology, have been used to modify proteins to enhance their functionality (Karabulut et al. 2024). HPH is a process in which a liquid is forced to pass through a narrow gap valve and, under the influence of several physical factors such as high pressure, turbulence, shear stress, cavitation, and impingement, promotes the dispersion of aggregates, changing the structure of the protein (Chen et al. 2017). US, with a frequency at a range of 20–100 kHz and a power of 10–1000 W/cm, is a processing technology that affects protein structures through forces like acoustic force, cavitation, shockwave, and micro‐jets (Zhang et al. 2021). HHP is a nonthermal technology in which liquid is typically used as a pressure transfer medium, and pressure is applied in the range of 200–1000 MPa at room temperature, resulting in the modification of protein structure due to generating strong mechanical stress and frictional heat (Dehnad et al. 2024; Jiang et al. 2023). During the protein modification by these techniques, non‐covalent interactions between proteins are disrupted, the size of protein aggregates is reduced, the formation of soluble aggregates from insoluble protein aggregates is promoted, and the spatial structure of the protein is unfolded to expose some reactive groups like free sulfhydryl (‐SH) and hydrophobic groups resulting in improved solubility with foaming and emulsifying properties (Yan et al. 2024).

Several types of research have been reported on the structural and techno‐functional modification of a plant‐based protein by HPH (Ma, Zhang, et al. 2023; Zhao et al. 2023), US (Du et al. 2022; Zhao, Liu, et al. 2022) and HHP (Kalayci et al. 2023; Yi and Liu 2023; Zhang, Zhang, et al. 2022). However, based on the mechanisms of HPH, US, and HHP, they are different, so the change of structural and functional properties of proteins modified by them might differ. Therefore, this work is designed to investigate the effect of HPH, US, and HHP on the structural and techno‐functional properties of sesame protein isolate from sesame cake as a by‐product. Selecting an appropriate modification method will enable the food industry to produce proteins with optimal techno‐functional properties for targeted food applications.

## 2 Materials and Methods

### 2.1 Materials

White sesame seeds with total solid and protein contents of 95.08% and 38.07%, respectively, were supplied by Aslan sesame and tahin food Co. Ltd. (Eskisehir, Turkey) and the seeds were stored at 4°C until used for protein extraction. All reagents and chemicals utilized in this study were purchased from Sigma‐Aldrich (Saint Louis, USA).

### 2.2 Preparation of Sesame Protein Isolate

After removing oil from sesame seed by cold‐press extraction, sesame protein isolate was prepared from sesame cake, as described (Gul et al. 2023), based on the method involving alkaline extraction and acid precipitation. The sesame cake was finally ground into small particles using a commercial blender and sieved at 0.25 mm. The resulted sesame cake powder was used for protein extraction. The obtained sesame powder was mixed in distilled water at pH 10 for 1 h in a powder/water ratio of 1:9 (g/mL). The mixture was centrifuged (9000 rpm for 10 min at 4°C) to separate soluble proteins from insoluble parts. After centrifugation, the supernatant was collected in a beaker, the pH was adjusted to 4.5 with 1 M HCl, and stirred continuously for 30 min, allowing the proteins to precipitate at isoelectric pH. The mixture was centrifuged under the same conditions as before, and the obtained pellet, which included precipitated proteins, was collected, frozen at −24°C for 24 h, and freeze‐dried with a freeze dryer (Teknosem, Toros TRS‐4/4, Turkey) at −56°C at 10^−3^ mbar pressure. Freeze‐dried protein, which was ground into a powder using an electric powder grinder, was used as a control sample. The resulting sesame protein isolate, with protein content of 90.33%, was stored in glass bottles at 4°C until use. The protein content of the isolate was determined in duplicate by the Kjeldahl method with a nitrogen conversion factor of 6.25.

### 2.3 Modification of Sesame Protein Isolate

The sesame protein suspension was prepared by dispersing the protein in distilled water to a concentration of 4 g/100 mL. After the pH of the suspension was adjusted to 7.0 with 1 M NaOH, this mixture was stirred for 90 min. The protein suspension samples were treated using HPH, US, and HHP.

Protein modification by the HPH was done in the manner previously mentioned by Baskinci and Gul (2023). To achieve this, a high‐pressure homogenizer (Panda PLUS, Italy) operating at 100 MPa for 1 cycle was applied to the protein suspension. Prior to homogenization, the sample temperature was measured to be 7°C; however, following homogenization, temperature readings of approximately 28°C were noted.

Protein modification by the US was done in the manner previously mentioned (Gul et al. 2023). Using an ultrasonic processor (JP Selecta, Spain) with a titanium probe with a 0.67 cm diameter, the protein solution was ultrasonically treated at a constant frequency of 20 kHz. The samples were subjected to a 6‐min treatment at a 95% amplitude ultrasonic power. Ice cassettes were utilized to keep the temperature below 40°C during the procedure.

Protein modification by the HHP was done in the manner previously mentioned (Gul et al. 2023). To achieve this, the protein suspension (0.5 L) was vacuum conditioned in polyethylene/polyamide bags and then placed in an HHP device (Avure Technologies Inc., USA). After the pressure cell was filled with pure water, pressure was applied for 5 min at 400 MPa at a pressure increase rate of 100 MPa/min. At the end of the process, the samples were removed by reducing the pressure of the sample chamber at a rate of 250 MPa/min.

Following protein modification using HPH, US, and HHP, protein suspensions were freeze‐dried for 48 h, crushed into powder, kept in airtight bags, and stored at 4°C for further analysis. The untreated protein isolate was used as a control. Each modification treatment by HPH, US, and HHP was carried out in triplicates.

### 2.4 Particle Size Distribution and Zeta Potential

The particle size distribution of untreated and modified sesame protein samples was determined using a laser diffraction particle size‐measuring device (Malvern Instruments, Mastersizer 3000 model, England). Sesame protein suspensions prepared at a specific concentration (4 mg/mL) were diluted with ultrapure water, and particle size analysis was performed at 25°C.

The zeta potential of untreated and modified sesame protein was measured by dynamic laser light scattering (Zetasizer Nano ZS90, Malvern Instruments, Worcestershire, UK). Freeze‐dried protein samples were suspended in ultrapure water (1% w/w) and placed in a cuvette where laser light set at an angle of 173° was scattered by the particles. For zeta potential measurements, the suspensions were diluted at a ratio of 1:100 (v/v) and the measurement was carried out at 25°C with the refractive index value fixed at 1.33.

### 2.5 Fourier Transform Infrared Spectra (FTIR)

FTIR analysis was used to detect possible changes in the secondary structures of sesame protein modified by nonthermal processing techniques. For this purpose, 3 mg untreated or modified protein isolate was weighed, mixed with a certain amount of potassium bromide, and scanned using an infrared spectrophotometer (Brüker Alpha‐II, Germany). The measurement range was set at 4000–400 cm^−1^ with a resolution of 4 cm^−1^ and scan times of 32. Additionally, the amide I region (1600–1700 cm^−1^) was subjected to a deconvolution procedure by using OriginLab software (OriginPro 9.1, USA), which determined the overlapped sub‐peaks related to secondary structures (α‐helix, β‐sheet, β‐turn, and random coil) by the secondary derivatives of the peaks (Parlak et al. 2024).

### 2.6 UV Spectra

The sesame protein sample was diluted with phosphate buffer to 0.5 mg/mL, and the UV absorption spectrum was analyzed using a UV–Vis spectrophotometer (Shimadzu, Japan). The solutions were scanned at a wavelength range of 200–400 nm at the medium scanning speed with a resolution of 0.5 nm at 25°C with a 1 cm path‐length quartz cell. The second derivative (*d* ^2^ *A*/*d* ^2^ *λ*) of the UV spectra was taken by using OriginLab software (OriginPro 9.1, USA), and the second derivative UV spectra were obtained (Zhao et al. 2023).

### 2.7 Differential Scanning Calorimetry (DSC)

Thermal characteristics of untreated and modified sesame protein were determined using a differential scanning calorimeter (DSC 1 STAR System, Mettler Toledo, Switzerland). An amount of 5 mg of protein samples was accurately added into aluminum pans, which were hermetically sealed afterward. An empty hermetically sealed aluminum pan was considered as the reference. The sample pans were allowed to stand for 1 h at 20°C to equilibrium and then heated from 20°C to 250°C at a rate of 10°C/min in nitrogen flow (10 mL/min). The transition temperatures (the onset temperature (*T*_onset_), the denaturation temperature (*T*_d_), and the end temperature (*T*_end_)) and the enthalpy changes of the endotherm (Δ*H*) of samples were calculated from the corresponding areas of DSC thermograms with the Mettler STARe Evaluation Software (Mettler Toledo, Switzerland).

### 2.8 Turbidity

The turbidity of untreated and modified sesame protein was determined by the method reported by Zhao et al. (2023). The protein sample was dispersed in phosphate buffer to obtain a 2 mg/mL protein solution and magnetically stirred for 60 min at 25°C. Then, the absorbance of the sample, which was performed to evaluate protein turbidity, was read at a 600 nm wavelength with a UV–Vis spectrophotometer (Shimadzu, Japan).

### 2.9 Free Sulfhydryl Group (— SH)

The free —SH group content of samples was measured according to the methodology described by Hu et al. (2013) using Ellman's reagent. Briefly, sesame protein was mixed with tris‐glycine buffer solution (containing 0.086 M tris‐(hydroxymethyl)aminomethane, 0.09 M glycine, and 4 mM ethylenediaminetetraacetic acid disodium salt) at a protein concentration of 0.2%. The mixture was stirred for 60 min at room temperature and then centrifuged at 8000× *g* for 15 min at 4°C. A 3 mL aliquot of the supernatant was mixed with 0.03 mL of Ellman's agent (4 mg/mL, dissolved in tris‐glycine buffer). After the mixture was kept in the dark at 20°C for 15 min, free —SH groups were determined by reading the absorbance at 412 nm wavelength on a spectrophotometer (Shimadzu, UV‐1800 240V, Japan). The tris‐glycine buffer was used instead of protein solutions as a blank. The free —SH content was calculated with the following formula.

(1) $$\text{Free}\;\mathrm{SH}\;\left(\mu \frac{\mathrm{mol}}{g}\right)=\left(73.53\times A_{412}\times D\right)/C$$

where, 73.53 = $10^{6}/\left(1.36\times 10^{4}\right)$ is Ellman's reagents molar extinction coefficient; *A*_412_ is the absorbance at 412 nm; C is the protein concentration of the solution (mg/mL); *D* is the dilution factor.

### 2.10 Protein Solubility

The protein solubility of untreated and modified sesame protein samples was determined according to the method reported by Klompong et al. (2007). For this purpose, freeze‐dried sesame protein samples were suspended using distilled water (1%, w/w) and mixed for 1 h at room temperature, then centrifuged at 8000 rpm for 15 min at 4°C (Nüve NF‐800R, Turkey). The separated supernatant was mixed with Biuret reagent in a one‐to‐one ratio, and the mixture was read on a spectrophotometer (Shimadzu, UV‐1800 240 V, Japan) at a wavelength of 500 nm. The protein content was evaluated using the standard curve obtained from bovine serum albumin solutions (*R* ^2^ = 0.9989), and the percent solubility was determined with Equation (2).

(2) $$\text{Solubility}\;\left(\%\right)=\frac{\text{Protein content in supernatnat}}{\text{Total protein}}\times 100$$

### 2.11 Emulsifying Properties

Emulsion activity index (EAI) and emulsion stability index (ESI) were taken into account in determining the emulsifying properties of untreated and modified sesame protein samples (Ogunwolu et al. 2009). Powdered protein sample (0.3 g) was suspended using 30 mL of distilled water, and 10 mL of sunflower oil was added to the suspension. Then, the mixture was homogenized using Ultra‐Turraks (Daihan, South Korea) for 1 min at 15,000 rpm. Immediately after the emulsion formed, a 50 μL aliquot of the emulsion sample was diluted with 5 mL of 0.1% sodium dodecyl sulfate, and the absorbance of the mixture was measured using a UV spectrophotometer at a wavelength of 500 nm. The same process was repeated 10 min after the emulsion formed. EAI and ESI were calculated using Equations (3) and (4), respectively.

(3) $$\mathrm{EAI}=\frac{2\times 2,303\times A_{0}}{0,25\times m}$$

(4) $$\mathrm{ESI}=\frac{A_{10}\times ∆t}{∆A}$$

where, *A*_0_ and *A*_10_ are the absorbance at 0 and 10 min, m is the weight of protein, ∆*t* is 10 min and ∆*A* is the difference between the initial and 10 min later absorbance value.

### 2.12 Oil Holding Capacity

The oil holding capacity of untreated and modified sesame protein samples was determined according to the method suggested by Ogunwolu et al. (2009). Briefly, 0.1 g of protein isolate was weighed into the test tube, mixed with 10 mL of sunflower oil, and then centrifuged at 8000 rpm for 10 min to remove the supernatant. The tubes were kept at a 45° angle for 20 min, and the accumulated oil was poured out. Then, the tubes were weighed, and the amount of bound oil was determined based on the weight difference (Equation 5).

(5) $$\text{Oil holding capacity}=\frac{W_{2}-W_{1}}{W_{0}}$$

where *W*_0_ (g) is the weight of the protein, *W*_1_ (g) is the weight of the test tube and sample, and *W*_2_ (g) is the weight of the test tube and precipitate.

### 2.13 Statistical Analysis

All the measurements were done in triplicates. The results were expressed as the mean ± standard deviation. Data were analyzed statistically by one‐way analysis of variance (ANOVA) with a 95% confidence limit, and Duncan's multiple comparison test was used to compare means by SPSS 21.0 (IBM, New York, USA). Origin 2021 software package (OriginLab Corporation, Northampton, USA) was used for principal component analysis and plotting.

## 3 Results and Discussion

### 3.1 Particle Size and Zeta Potential

Particle size and zeta potential results of unmodified (control) and sesame protein samples modified with different methods are given in Table 1. The average particle size of the control sample was relatively large (65.73 μm), and a significant decrease was detected due to the modification process with different methods (*p* < 0.05). Similar results have been revealed in various studies in the literature, and Yan et al. (2024) reported that the particle size of the pea protein modified by HPH and US decreased approximately four times after the modification. This may be due to the shear, cavitation, and turbulence effects on protein molecules during modification by physical methods such as HPH, US, and HHP (Chen et al. 2017; Ding et al. 2022; Jiang et al. 2017). The average particle size of modified sesame protein samples was measured between 1.48 and 7.44 μm. While the lowest value was determined in the samples treated with HPH, the highest value was found in the protein samples modified with the HHP method. The high‐energy shear force and turbulence produced by the cavitation effect reduce or may even help eradicate the aggregation of proteins due to the disruption of hydrogen bonds, hydrophobic interactions, and electrostatic interactions (Gao et al. 2022; Wu et al. 2020), resulting in narrower causes of particle dispersion and a smaller average particle size.

**TABLE 1:** Average particle size and zeta potential values of unmodified and modified sesame protein isolates.

Sample	Particle size (μm)	Zeta potential (mV)
Control	65.73 ± 6.64^a^	−24.57 ± 0.62^c^
HPH	1.48 ± 0.43^d^	−42.8 ± 0.18^a^
US	4.54 ± 0.36^c^	−37.6 ± 0.47^b^
HHP	7.44 ± 1.29^b^	−36.8 ± 0.36^b^

*Note:* Different superscripted letters of different columns represent significant differences at *p* < 0.05. Control, unmodified sesame protein isolate; HHP, high hydrostatic pressure treated sesame protein isolate; HPH, high‐pressure homogenization treated sesame protein isolate; US, high intensity ultrasound treated sesame protein isolate. Values are given as mean ± SD from triplicate determinations.

Zeta potential is measured by the dynamic light scattering method by evaluating the electrophoretic mobility of particles, and electrophoretic mobility data is converted to zeta potential with the Henry equation (Dehnad et al. 2023; Manassero et al. 2018). The strength of the electrical charges on colloidal surfaces defines the zeta potential, a substantial measure of particle stability (Dehnad et al. 2023). Ionization of different amino acid residues causes the effective charge on the protein particle, which is influenced by pH, ionic strength, and the accumulation of surfactants at the interface. The magnitude of the absolute value of the zeta potential in the solution system indicates the repulsion force between molecules, which means that the tendency to aggregate in the system will decrease (Gul et al. 2023). As shown in Table 1, the zeta potential values of sesame protein isolates were measured as negative charge, indicating that there are more negatively charged amino acids on the surface of sesame protein. In the control sample, the zeta potential was determined as −24.57 mV, and the modification process with different methods led to an increase in zeta potential, which was determined between −36.8 and − 42.8 mV. The increase in zeta potential with the nonthermal treatments indicates that it will play an essential role in increasing the stability of the protein solution due to good electrostatic repulsion (Gul et al. 2023). The increase in zeta potential is associated with the increase in surface area in response to decreasing particle size, the movement of polar groups in the inner regions of the protein molecule towards the surface of the protein molecules, and the change of amino acids on the protein surface (Tang and Ma 2009). Therefore, there is more net charge on the modified protein surface, and electrostatic interactions form more tightly bound complexes, enhancing the distribution and stability of the complex (Wang et al. 2021). Similar results regarding the increase of zeta potential with physical methods applied in protein modification exist. It has been reported that US (Gul et al. 2023) and HPH (Baskinci and Gul 2023) applications cause an increase in the zeta potential values of sesame protein isolate, and high hydrostatic pressure application causes an increase in the zeta potential values of sweet potato protein (Zhao et al. 2018).

### 3.2 FTIR

**FIGURE 1:**
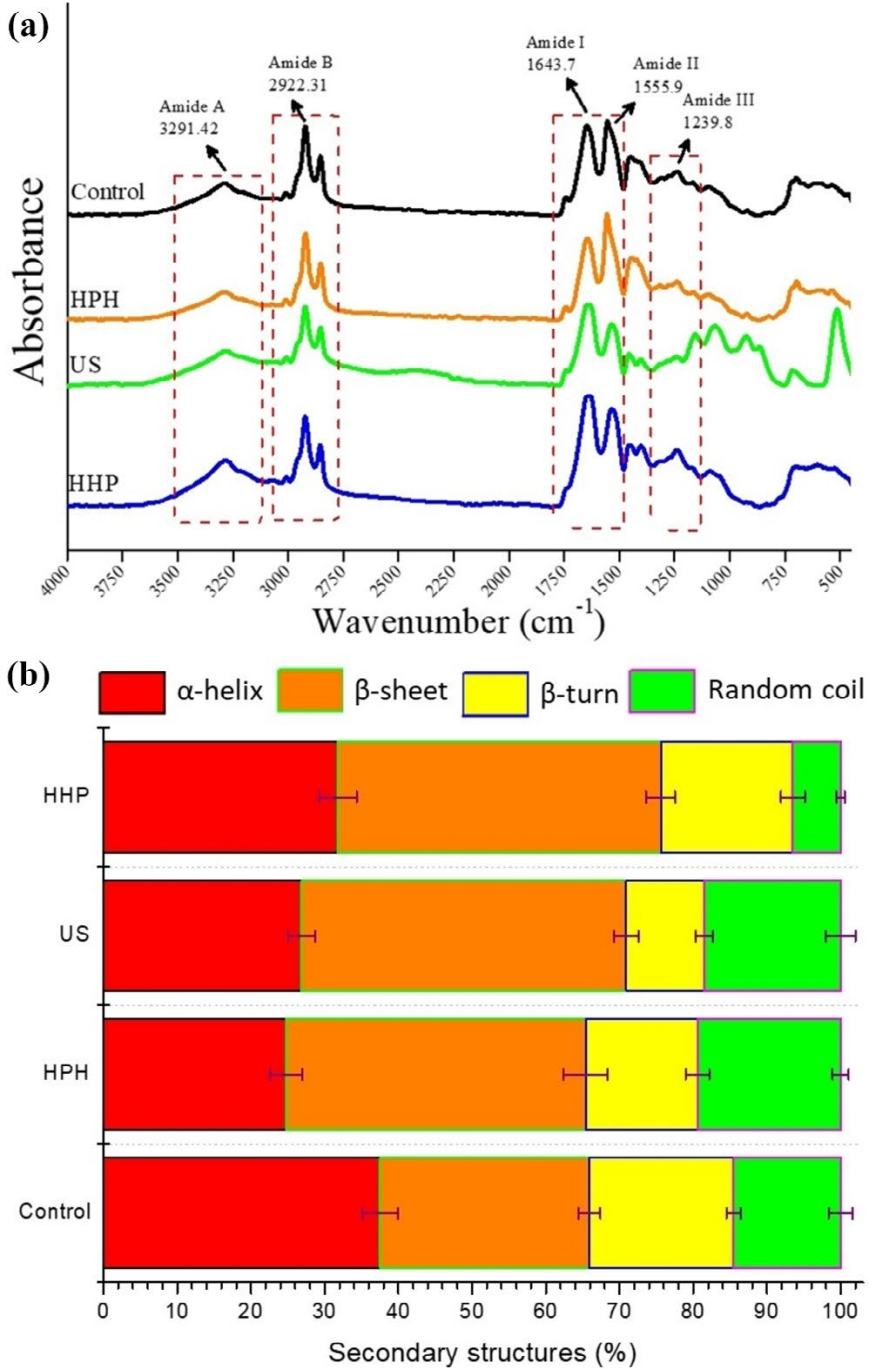
FTIR spectra (a) and second derivative (b) of FTIR spectra of unmodified and modified sesame protein isolates (Control, unmodified sesame protein isolate; HHP, high hydrostatic pressure treated sesame protein isolate; HPH, high‐pressure homogenization treated sesame protein isolate; US, high intensity ultrasound treated sesame protein isolate).

FTIR is one of the powerful techniques used to analyze the secondary structure of proteins (De Meutter and Goormaghtigh 2021). It provides information about the secondary structures of proteins through wavelength shifts in Amide I, Amide II, and Amide II regions. The amide I region (1700–1600 cm^−1^) is controlled by 80% C=O and 10% C—N stretching vibrations and is the most sensitive band to changes in the secondary structure of proteins. The Amide II region (1580–1480 cm^−1^) consists of 60% N—H bending, 30% C—N, and 10% C—C stretching, and the Amide III region (1400–1200 cm^−1^) contains more complex bonds, which are associated with C—N stretching and N—H rotational vibrations (Chen et al. 2018; Sadat and Joye 2020). The FTIR spectra recorded for control and modified sesame protein isolate samples are illustrated in Figure 1a. For all sesame protein samples, major absorption bands were detected in the spectrum at 3291 cm^−1^ for Amide A (OH‐stretching), 2922 cm^−1^ for Amide B, 1643 cm^−1^ for Amide I, 1555 cm^−1^ for Amide II, and 1239 cm^−1^ for Amide III. Considering the overall FTIR profile, including the absorption peak position and their relative intensities, which was in agreement with previous reports (Baskinci and Gul 2023; Gul et al. 2023), while there was no difference in peak position among the protein samples, the intensity of bands changed with modification comparing the control sample. The changes in band intensity after modification with HPH, US, and HHP are probably due to the breaking or emergence of some H and/or covalent bonds in the protein structure due to the physical alterations that take place during modification (Baskinci and Gul 2023).

Since the Amide I region consists of α‐helix (1650–1658 cm^−1^), β‐sheet (1610–1640 cm^−1^), β‐turns (1660–1695 cm^−1^) and random coils (1640–1650 cm^−1^) is usually used to analyze the composition of the secondary structure of the protein, the deconvolution in this region was conducted, and the percentage of each structural component is shown in Figure 1b. Generally, α‐helix structures are formed mainly through intramolecular hydrogen bonds between carbonyl oxygen (—CO) and amino hydrogen (NH—) groups, which are buried in the protein interior site, while interchain hydrogen bonding between polypeptide chains stabilizes β‐sheets. Also, weak hydrogen bonds form β‐turn structures, and random coils correspond to the unfolded conformation, which is related to protein flexibility (Chen et al. 2017; Li and Arakawa 2019). As seen in Figure 1b, unmodified sesame protein contained 37.52% α‐helix, 28.39% β‐sheet, 19.58% β‐turn, and 14.49% random coil, and in general, α‐helix content was dominant in sesame protein. However, the α‐helix content in sesame protein decreased with the modification applied, whereas the β‐sheet content increased and became dominant. Consistent with our findings, the decreasing α‐helix content and increasing β‐sheet were also stated by Zhao et al. (2023) for quinoa protein treated with HPH, by Zhao, Liu, et al. (2022) for potato protein isolates treated with US, and by Luo, Yang, et al. (2022) for quinoa protein treated with HHP, indicating that protein structures could unfold as a result of all modification treatments. The cavitation effect and mechanical forces during modification processes by HPH, US, and HHP cause a partial opening of the α‐helix region, promoting a decrease in α‐helix content and an increase in β‐sheet content (Kang et al. 2016). Moreover, the β‐sheet content rises, and the α‐helix content falls when hydrophobic regions, such as sulfhydryl and hydrophobic groups of proteins, are exposed (Zhang et al. 2014). The creation of intermolecular β‐sheet structures is facilitated by a rise in protein–protein interactions, as demonstrated by the increase in β‐sheet content (Chen et al. 2017). In addition, it has been reported that the increasing β‐sheet content has a positive effect on the generation of protein gels (Choi and Ma 2007), increasing the emulsifying ability and stability and improving the strength of protein against flocculation and creaming (Yang et al. 2018).

A nonsignificant decrease in the β‐turn content was observed in the sample treated with HPH. However, a significant increase was detected in the samples treated with US and HHP. An increase in β‐turn content is expected to alter the solution's potential value, which is supported by the findings of the zeta potential experiment because it is typically present on the surface of proteins with polar and charged amino acid residues (Ma, Xu, et al. 2023). This approach was not accepted for the HPH‐modified protein sample with the highest zeta potential. On the other hand, while a decreasing trend was observed in the random coil content of sesame protein modified by HPH, a decrease was observed because of the application of HHP and US. The reduction in random coil content in the modified sample indicated that the protein had changed from a disordered structure to an ordered structure (Zhang, Wang, et al. 2022). The FTIR results revealed that HPH, US, and HHP treatments alter the secondary structures in sesame proteins, causing the unfolding of proteins, the transformation of insoluble clumps into soluble ones, and the release of hydrophobic protein residues. However, these changes vary with different modifications, as reported previously by Yang et al. (2018).

### 3.3 UV Spectra

**FIGURE 2:**
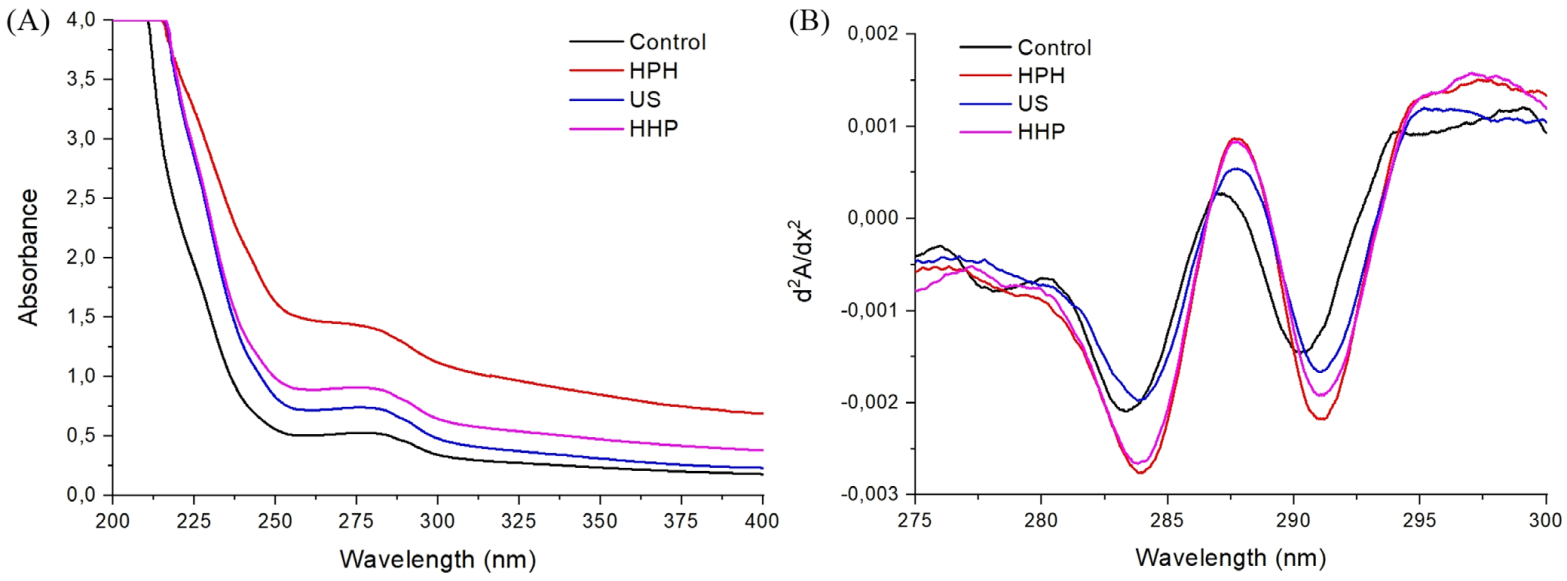
UV spectra (a) and second‐derivative spectrum (b) of unmodified and modified sesame protein isolates (Control, unmodified sesame protein isolate; HHP, high hydrostatic pressure treated sesame protein isolate; HPH, high‐pressure homogenization treated sesame protein isolate; US, high intensity ultrasound treated sesame protein isolate).

UV absorption spectroscopy is a widespread method used to explore the transformation in the tertiary structure of proteins and changes in the aromatic side chains of tyrosine (Tyr), phenylalanine (Phe), and tryptophan (Trp) residues (Yan et al. 2024; Zhao, Xie, et al. 2022). The UV spectra and second‐derivative spectrum of the control and modified sesame protein isolates are shown in Figure 2. As shown in Figure 2a, a characteristic peak at 275–280 nm was observed due to the combined impact of Tyr (275 nm), Trp (279 nm), and Phe (257 nm) (Mozafarpour et al. 2022). The absorption intensity of the modified samples was higher than that of the control sample, indicating that multiple forces during the modification with different nonthermal techniques can ultimately unfold the protein molecules and, thereby, more buried hydrophobic amino acids such as Trp, Tyr, and Phe were transferred to the surface of the protein molecules (Mozafarpour et al. 2022). According to the previous studies (Baskinci and Gul 2023; Gul et al. 2023), buried hydrophobic groups can be exposed to protein surfaces after modification by nonthermal treatments. As shown in Figure 2, the absorbance value of the sesame protein treated with HPH was considerably higher than that of treated with other techniques, and the action of US was the weakest.

Second‐derivative fitting was used to examine the microenvironmental changes of the Trp and Tyr residues because of its exceptional ability to separate the distinct contributions of aromatic side chains from overlapping bands in the typical spectrum; it has been demonstrated to be a proper analytical technique for detecting conformational changes in proteins (Xu et al. 2012). All protein samples showed two prominent peaks at 287 and 294 nm and two troughs at 282 and 291 nm (Figure 2b), consistent with the findings for quinoa protein by Zhao et al. (2023). According to Wang et al. (2020), the combined contributions of the Trp and Tyr residues are responsible for the peak at 287 nm, while Trp alone is responsible for the peak at 296 nm. Compared to the control sample, the peak positions of second derivative spectra shift to the red region (longer wavelengths). This red region could be explained by greater unfolding of the peptide chain and exposing the hydrophobic amino acid residues from the protein interior to the exterior, enhancing the intermolecular surface hydrophobicity of protein and resulting in change of the protein tertiary structure (Zhao et al. 2023).

### 3.4 Thermal Properties

The thermal properties of proteins play an essential role in their functionality and consequently, their usability in food systems, and determine the suitability of the protein for different applications in food systems involving some heating steps. The DSC data, including onset (*T*_onset_), denaturation (*T*_d_), and end temperature (*T*_end_), as well as enthalpy changes of the endotherm (Δ*H*), obtained for untreated and modified sesame protein isolates are presented in Table 2. All protein samples exhibited endothermic peaks, probably due to the non‐covalent interactions comprising hydrogen bonds and van der Waals interactions in protein isolates (Youshanlouei et al. 2024). The temperature for the control sample was determined as 65.55°C and no major difference occurred with the modification by HPH, US, or HHP. Moreover, considering *T*_d_, which indicates the thermal stability of protein concerning the temperature at which protein denaturation occurs, the control sample showed an endothermic peak at 97.87°C, while the peak of the modified sesame protein samples had a similar endothermic peak but slightly lower. Similarly, studies conducted on the faba bean isolate treated with US (Badjona et al. 2024), chickpea protein isolate treated with HPH up to 120 MPa (Huang et al. 2022), and sweet potato protein isolate treated with HHP (Sun et al. 2014), have observed a decrease in their thermal transition peak temperature after modification. The cause of the somewhat lower denaturation temperature could be the physical structural change brought on by the nonthermal treatments. The temperature stability and hydrogen bond breakage that occur in tertiary and quaternary structures are reflected in *T*_d_. As a result, the decrease in *T*_d_ promoted by the nonthermal treatments indicates a disruption in the tertiary structure of sesame protein isolate, thus the flexibility of the protein may have increased (Sun et al. 2014). It has been claimed that the enthalpy change (Δ*H*) was used to determine the energy required to dominate non‐covalent interaction, which is correlated with the extent of protein unfolding (Saatchi et al. 2020; Sun et al. 2014). As shown in Table 2, a Δ*H* value of 67.71 J/g protein was found for the untreated sample, and nonthermal treatments led to a significant decrease in the Δ*H* to 56.06 J/g protein with a residual enthalpy of 82.79% (*p* < 0.05). However, no significant difference in Δ*H* was detected among the modified samples (*p* > 0.05). The reduction was probably due to the fact that after nonthermal treatment with HPH, US, and HHP, there was a partial loss in the ordered protein structure (Sun et al. 2014), as confirmed by the FTIR observations.

**TABLE 2:** Thermal properties of unmodified and modified sesame protein isolates.

Sample	*T*_onset_ (°C)	*T*_d_ (°C)	*T*_end_ (°C)	Δ*H* (J/g protein)	Residual enthalpy (%)
Control	65.55 ± 0.58^b^	97.87 ± 0.81^a^	135.13 ± 0.46^a^	67.71 ± 1.18^a^	100
HPH	65.2 ± 0.41^b^	97.11 ± 0.38^a^	131.97 ± 0.71^b^	56.06 ± 0.73^b^	82.79
US	68.8 ± 0.62^a^	96.69 ± 0.54^a^	132.82 ± 0.55^b^	56.61 ± 0.94^b^	83.61
HHP	66.32 ± 0.29^b^	96.02 ± 0.71^a^	133.84 ± 0.36^ab^	57.07 ± 1.12^b^	85.76

*Note:* Different superscripted letters of different columns represent significant differences at *p* < 0.05. Δ*H*, the enthalpy changes of the endotherm; Control, unmodified sesame protein isolate; HHP, high hydrostatic pressure treated sesame protein isolate; HPH, high‐pressure homogenization treated sesame protein isolate; *T*_d_, thermal denaturation temperature; *T*_e_, end temperature of denaturation; *T*_onset_, onset temperature of denaturation; US, high intensity ultrasound treated sesame protein isolate. Values are given as mean ± SD from duplicate determinations.

### 3.5 Turbidity

**FIGURE 3:**
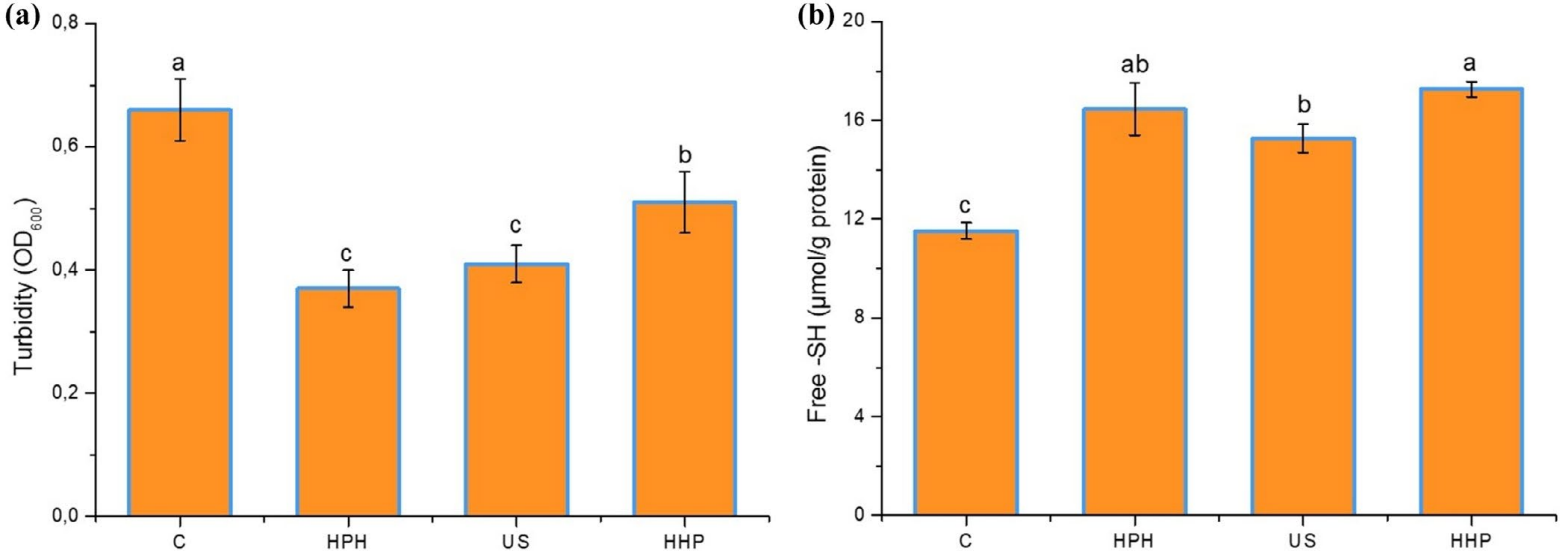
Turbidity (a) and Free —SH group content (b) of unmodified and modified sesame protein isolates (Control, unmodified sesame protein isolate; HHP, high hydrostatic pressure treated sesame protein isolate; HPH, high‐pressure homogenization treated sesame protein isolate; US, high intensity ultrasound treated sesame protein isolate).

Turbidity of the protein solution is related to both the number and the size of the soluble protein aggregates, so it is used to determine the degree of protein aggregation. As shown in Figure 3a, the highest turbidity (0.665) was detected in the control sample, and modification with HPH, US, and HHP eventuated a significant decrease in turbidity of sesame protein (*p* < 0.05). The same results were obtained by Zhao et al. (2023) and by Ma, Xu, et al. (2023) in studies on quinoa and housefly larvae proteins, respectively. A decrease in turbidity is generally correlated to a reduction in particle size due to the multiple forces generated during modification (Ma, Zhang, et al. 2023). Moreover, as a result of the modification, the particle size decreased, the particle surface area and water solubility increased, and the zeta potential increased, which caused more electrostatic repulsion between protein molecules, resulting in a homogeneous and stable protein solution and lower turbidity was measured (Zhao et al. 2023). The highest decrease (42.35%) in turbidity value was detected in the sample modified with HPH, while the lowest decrease (21.29%) was observed in the sample modified with HHP. The changes in turbidity of the sesame protein solution conformed to the findings of the particle size and zeta potential.

### 3.6 Free —SH Groups

Changes in free —SH groups involved in the secondary and tertiary structure of proteins directly affect the conformational stability of the protein molecules, which in turn affects the functionality of the protein (Kim and Shin 2022; Ma, Xu, et al. 2023). The free —SH group content of sesame proteins before and after nonthermal treatments is presented in Figure 3b. Unlike the control sample (11.53 μmol/g protein), the free —SH group contents of the nonthermal treated sample significantly increased to the peak value (17.93 μmol/g protein) (*p* < 0.05). Similar findings were reported with previous works using the nonthermal treatment on various plant protein samples including sesame protein isolates (Zhang et al. 2024), potato protein isolates (Zhao, Liu, et al. 2022), and rice bran protein isolates (Yi and Liu 2023). This augmentation could be ascribed to breaking disulfide bonds to form —SH groups and exposing —SH groups (Zhao, Liu, et al. 2022). The physical forces such as high pressure, shear stress, turbulence, cavitation, high shear forces induced by HPH, US, and HHP, and also some other factors like increasing temperature can lead to the unfolding of the protein, the splitting of intra‐ and intermolecular S—S bonds, and the exposure of the —SH groups (Xue et al. 2018). Among the thermal treatment groups, the HPH‐treated protein exhibited the highest free —SH groups (17.93 μmol/g protein), while the US treated sample was the lowest (14.73 μmol/g protein). Ma et al. (2024) also stated that US had a weaker effect on thefree —SH content than other nonthermal treatments. In contrast, compared to the untreated sample, Shi et al. (2020) discovered that the free‐SH content of the whey protein isolate increased by 42.27% at 120 MPa of the HPH pressure and by 90.60% under 600 W of the ultrasonic power. Our free —SH content findings were in keeping with changes in protein solubility results.

## 4 Protein Solubility

**FIGURE 4:**
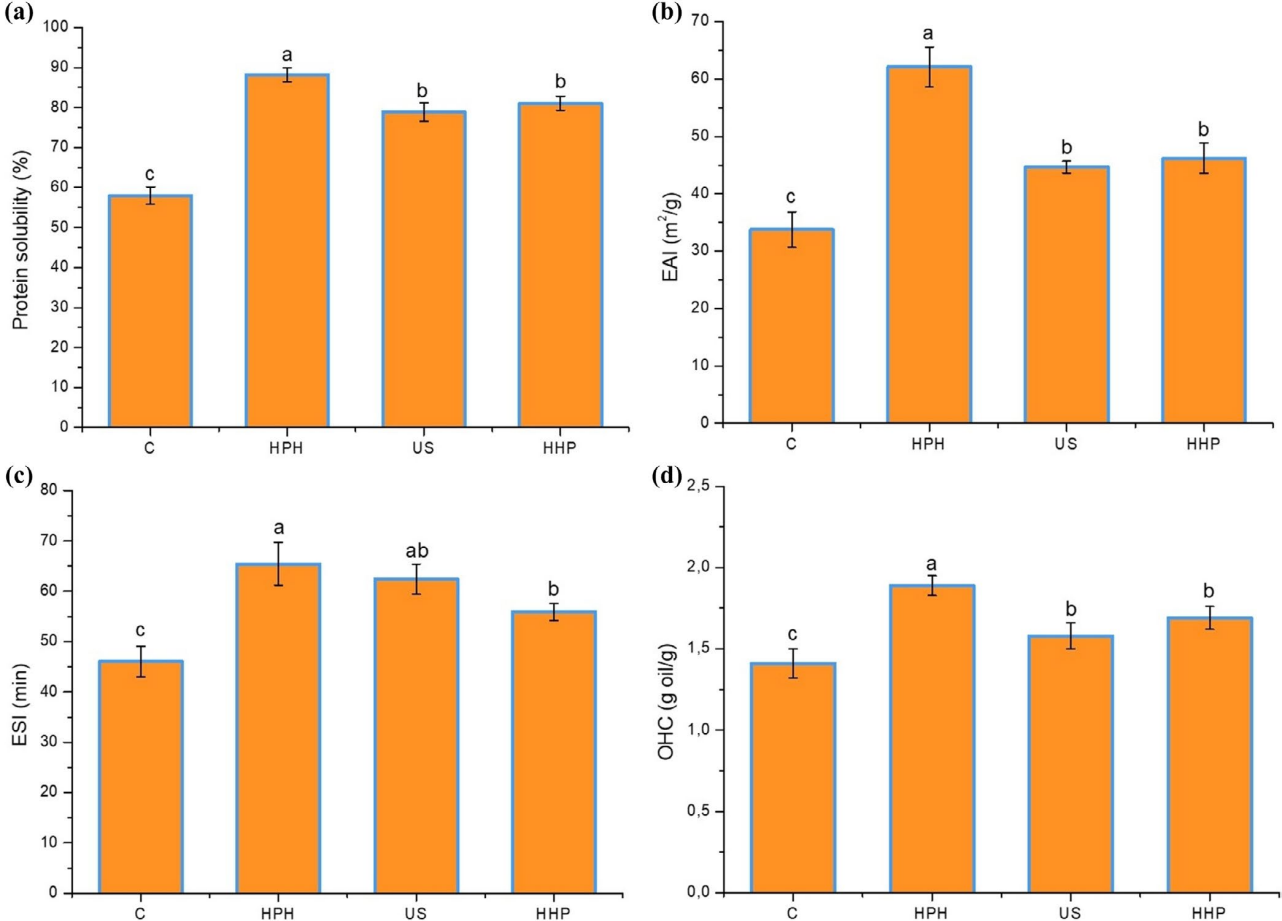
Protein solubility (a), EAI (b), ESI (c) and OHC (d) unmodified and modified sesame protein isolates (Control, unmodified sesame protein isolate; HHP, high hydrostatic pressure treated sesame protein isolate; HPH, high‐pressure homogenization treated sesame protein isolate; US, high intensity ultrasound treated sesame protein isolate).

Proteins are complex electrolytes with positive and negative charges that can be dispersed in water (Han et al. 2020). Solubility is a specific indicator of protein hydration. This is an effective indicator of protein conformational changes and aggregation properties and a positive parameter for the techno‐functional properties of the protein, such as foaming, emulsification, gelation, film formation, and viscosity (Jambrak et al. 2008). It plays a crucial role in developing new protein processing techniques and creating commercially valuable functional foods (Meng et al. 2019). Most plant proteins differ significantly from animal proteins and are generally lower in solubility. Although sesame protein has the potential to be used to improve various properties of foods due to some of its techno‐functional properties, its use is limited due to its low solubility (Baskinci and Gul 2023). The solubility results of control and sesame protein isolates modified with three different physical methods are given in Figure 4a. The protein solubility of the control sample was measured at 58.03%, and the applied modification process detected a significant improvement in the solubility. While the highest solubility value (88.18%) was reached with the application of HPH, the solubility of sesame protein after modification with US and HHP reached 78.92% and 81.09%, respectively. Similarly, Yan et al. (2024) reported that the solubility of pea protein after modification with HPH or US was three times higher than that of the control sample. Xue et al. (2018) reported that only the HHP application provided a significant improvement in the solubility of the myofibrillar protein, whereas US and HPH applications caused a decrease in protein solubility. It is estimated that these differences may be due to the different conformational properties of the protein used in the study, different ionic strengths in the environment, solution pH values, different extraction methods used in protein extraction, and even modification process conditions. There are several reasons for the increase in protein solubility after modification, the first of which is that effects such as cavitation, high shear, and turbulence caused by the applied physical methods cause the reduction of larger protein aggregates into smaller aggregates, making them more soluble (Lan Luo, Cheng, et al. 2022; Tang et al. 2009). Second, mechanical forces and cavitation forces disrupt hydrogen and hydrophobic bonds, causing partial cleavage or opening of the protein and causing more surface area to interact with water molecules, resulting in the release of more hydrophilic amino acid residues (Jambrak et al. 2009; Tang et al. 2021). Thirdly, applying HPH, US, or HHP increases the exposure of positively charged groups on the protein surface, thus causing stronger electrostatic repulsion between protein molecules and further increasing protein solubility (Ong et al. 2022).

### 4.1 Emulsifying Properties and Oil Holding Capacity

**FIGURE 5:**
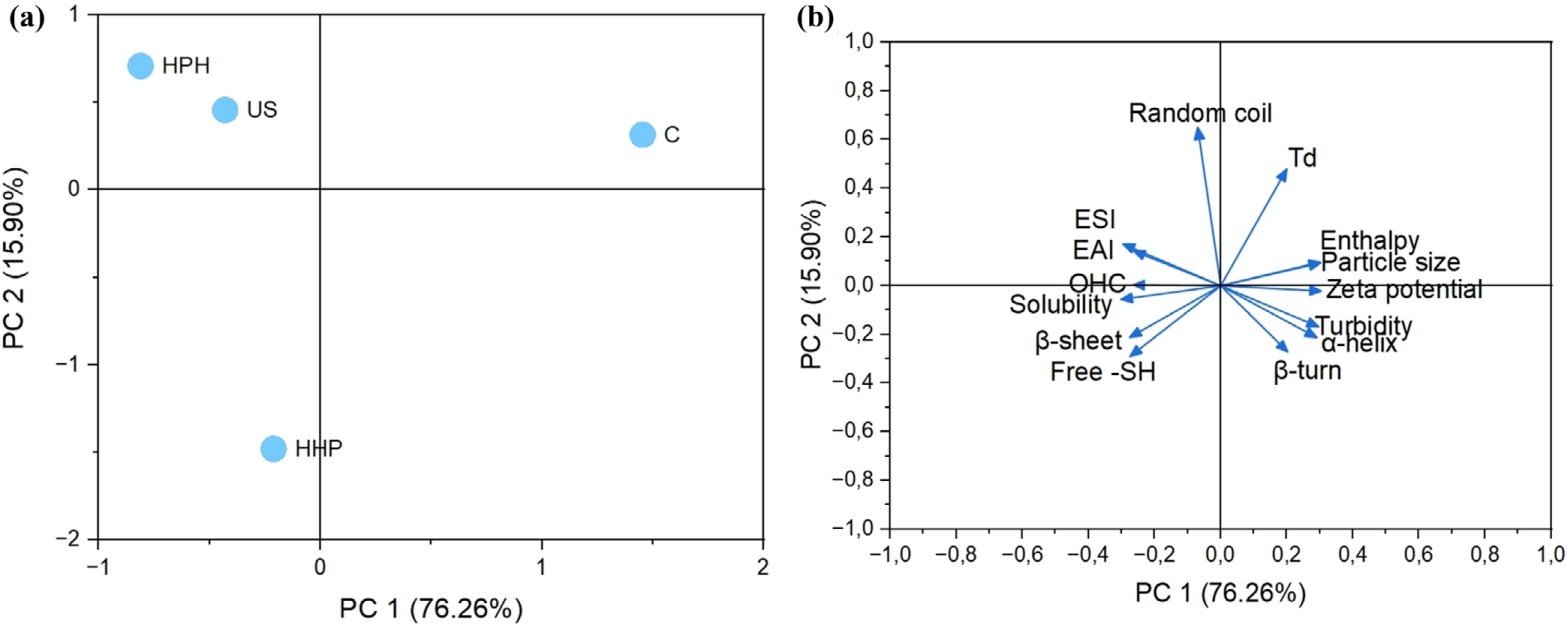
Principal component analysis (PCA) of structural, thermal and techno‐functional properties of unmodified and modified sesame protein isolates. (a) PCA Score plots; (b) PCA loading plots. HHP, high hydrostatic pressure treated sesame protein isolate; HPH, high‐pressure homogenization treated sesame protein isolate; US, high intensity ultrasound treated sesame protein isolate.

Proteins can contribute significantly to mixing two immiscible phases to form homogeneous emulsions in oil/water emulsion systems. The emulsification of proteins is evaluated according to the emulsion activity index (EAI) and emulsion stability index (ESI). EAI is widely used to compare the emulsifying abilities of proteins, and a high EAI value indicates that proteins can rapidly adsorb on the oil droplet surface, forming oil–water interfaces. ESI shows the ability of proteins to prevent the separation of the resulting emulsion; in other words, the ability to keep emulsions stable for a certain period (Gao et al. 2022). The change in the emulsifying properties of proteins is closely related to various factors such as structural properties, solubility, surface hydrophobicity, oil/water ratio, and environmental pH (Chen et al. 2019). Emulsification properties (EAI and ESI) of unmodified and modified sesame protein isolates with three different methods are given in Figures 4b and 5c, respectively. EAI and ESI values of the control sample were determined as 33.76 m^2^/g and 46.03 min, respectively. After modification of sesame protein, EAI and ESI values tended to increase significantly and were measured in the ranges of 44.65–62.09 m^2^/g and 55.91–65.43 min, respectively. While the highest EAI and ESI values were detected in protein samples modified with HPH, the slightest change in EAI value was observed after US and ESI value after HHP application. Increased EAI value suggests that proteins show higher emulsifying activity for the emulsion produced after modification, while higher ESI values indicate that these emulsions have higher stability during storage. Shi et al. (2020) reported that after modification of whey protein with HPH and US, the EAI values of protein samples improved by 8.54% and 7.63%, respectively, compared to the control. Another study reported that the EAI and ESI values of myofibrillar protein‐soybean oil emulsion were significantly enhanced with the application of HPH and US (Zhou et al. 2022).

EAI, one of the essential techno‐functional properties of proteins, generally depends on the structural properties of proteins, such as surface hydrophobicity and particle size. The smaller the protein particle size, the better the emulsification effect (Han et al. 2020). The increase in surface hydrophobicity after modification can increase the hydrophobic interaction between adjacent protein molecules at the interface, thus improving the emulsifying activity (Shen et al. 2017). In addition, increased protein solubility also plays a role in enhancing emulsion properties. Cavitation, high shear force, and turbulence that occur during modification by physical methods can intensify the particle size distribution of the protein and reduce the particle size, thus enabling the protein to be adsorbed more efficiently at the oil–water interface (Shevkani et al. 2015).

Oil holding capacity is the physical entrapment of oil, and the oil‐holding mechanism involves capillary interaction, allowing absorbed oil to be retained (Kinsella 1979). Hydrophobic proteins play the leading role in oil absorption. According to Sathe et al. (1982), oil holding capacity may be related to protein contents, protein types, and amino acid composition of proteins, especially hydrophobic residues that interact with hydrocarbon chains in oil molecules. Additionally, Kinsella (1979) demonstrated that more hydrophobic proteins showed superior holding to lipids, indicating that nonpolar amino acid side chains bind to the paraffin chains of oils. Therefore, the oil holding capacity of different protein isolates is affected by the protein particle size, content, composition, and structure of the protein component. Oil holding capacity is essential in increasing mouthfeel and maintaining or improving flavor (El Nasri and El Tinay 2007). For this reason, oil holding capacity is an issue that needs to be emphasized in many food applications such as bakery products, various food formulations, and meat substitutes, and plant‐based proteins with high oil holding capacity must have a high potential to be used in the food industry. The oil‐holding capacities of unmodified sesame protein and protein samples modified with HPH, US, and HHP are given in Figure 4d. The lowest oil holding capacity (1.41 g oil/g protein) was detected in the control sample. After modification of sesame protein with HPH, US, and HHP techniques, a significant increase in oil holding capacity was detected (*p* < 0.05), and the highest value was noted as 1.89 g oil/g protein in the sample modified with HPH. The increase in oil‐holding capacity is due to the partial dissolution of protein structures occurring during modification and the exposure of hydrophobic groups on the surface, promoting the formation of a network in a structure that traps oil droplets (Paglarini et al. 2019). Consistent with our results, Melchior et al. (2022) reported that HPH application had a positive effect on the oil‐holding capacity of pea protein. He et al. (2014) found that the oil‐holding capacity of peanut protein isolate after high‐pressure treatment was significantly higher than that of unpressurized peanut protein and commercial soybean isolates. Resendiz‐Vazquez et al. (2017) reported that the oil‐holding capacity of jackfruit ( *Artocarpus heterophyllus* ) seed protein isolates treated with ultrasound was higher compared to the untreated sample and increased from 1.92 g oil/g protein to 3.22 g oil/g protein.

### 4.2 Principal Component Analysis

The principal component analysis was employed to comprehensively assess the effect of different modification techniques on the observations of sesame seed protein isolate (Figure 5). The eigenvalues of the first two principal components (PC1 and PC2) among the major components were found to be more than 1 (10.67 and 2.23, respectively), which accounted for the observational difference among the samples. According to the results, the main components accounted for 99.4% of the cumulative variance contribution rate, which indicated a reliable separation/grouping. PC1 was the main factor affecting structural and functional characteristics, accounting for 76.26% of the overall variance. As seen in the score plot (Figure 5a), the control sample was significantly different from the modified protein samples, indicating that it had different structural and functional characteristics. The protein sample modified with HHP was separated from HPH and US modified proteins, demonstrating different modification techniques exerted different influences. PC1 showed positive interrelations with Td, enthalpy (ΔH), particle size, zeta potential, turbidity, α‐helix, and β‐turn of sesame protein isolate (Figure 5b), indicating that an increase in PC1 values corresponds to significant structural and thermal modifications. Meanwhile, random coil, EAI, ESI, solubility, β‐sheet, and free —SH content were regarded as characteristic indices of PC2, reflecting protein unfolding and enhanced functionality.

## 5 Conclusions

In this study, the sesame protein isolate was modified using nonthermal techniques, namely HPH, US, and HHP. After the modification of the sesame protein isolate with all treatments, the particle size, turbidity, α‐helix, and β‐turn of the sesame protein decreased, whereas the zeta potential, β‐sheet, random coil (except HHP), free —SH content, protein solubility, and techno‐functional properties such as EAI, ESI, and OHC increased considering the untreated protein sample. On the other hand, thermal analysis using DSC revealed minimal changes in the thermal characteristic profile of modified sesame protein isolates. Among the different modifications, more pronounced structural and molecular alterations in the protein sample treated with HPH were observed, resulting in further improved techno‐functional properties. Therefore, HPH is an effective technique to modify the structural and techno‐functional properties of sesame protein isolate, which provides new insights into the use of it as a functional ingredient or the development of plant‐based food products.

## Author Contributions

**Osman Gul:** conceptualization (lead), funding acquisition (lead), investigation (equal), methodology (equal), project administration (lead), supervision (lead), writing – original draft (equal), writing – review and editing (equal). **Melike Seyda Sahin:** data curation (equal), formal analysis (equal), writing – original draft (equal). **Abdullah Akgun:** data curation (equal), formal analysis (equal), investigation (equal), writing – review and editing (equal). **Latife Betul Gul:** data curation (equal), formal analysis (equal), investigation (equal), writing – review and editing (equal).

## Conflicts of Interest

We declare that we have no financial or personal relationships with other people or organizations that can inappropriately influence our work. All authors of this manuscript have directly participated in the program execution and/or analysis of this study. The content of this manuscript has not been copyrighted or published before. The content of this manuscript is not currently considered for publication elsewhere.

## Data Availability

All data generated and/or analyzed during this study are available from the corresponding author on reasonable request.
